# A pan-respiratory antiviral chemotype targeting a transient host multi-protein complex

**DOI:** 10.1098/rsob.230363

**Published:** 2024-06-19

**Authors:** Maya Michon, Andreas Müller-Schiffmann, Anuradha F. Lingappa, Shao Feng Yu, Li Du, Fred Deiter, Sean Broce, Suguna Mallesh, Jackelyn Crabtree, Usha F. Lingappa, Amanda Macieik, Lisa Müller, Philipp Niklas Ostermann, Marcel Andrée, Ortwin Adams, Heiner Schaal, Robert J. Hogan, Ralph A. Tripp, Umesh Appaiah, Sanjeev K. Anand, Thomas W. Campi, Michael J. Ford, Jonathan C. Reed, Jim Lin, Olayemi Akintunde, Kiel Copeland, Christine Nichols, Emma Petrouski, Ana R. Moreira, I-ting Jiang, Nicholas DeYarman, Ian Brown, Sharon Lau, Ilana Segal, Danielle Goldsmith, Shi Hong, Vinod Asundi, Erica M. Briggs, Ngwe Sin Phyo, Markus Froehlich, Bruce Onisko, Kent Matlack, Debendranath Dey, Jaisri R. Lingappa, Dharma M. Prasad, Anatoliy Kitaygorodskyy, Dennis Solas, Homer Boushey, John Greenland, Satish Pillai, Michael K. Lo, Joel M. Montgomery, Christina F. Spiropoulou, Carsten Korth, Suganya Selvarajah, Kumar Paulvannan, Vishwanath R. Lingappa

**Affiliations:** ^1^ Prosetta Biosciences, San Francisco, CA, USA; ^2^ Institute of Neuropathology, Heinrich Heine University, Düsseldorf, 40225 Germany; ^3^ Institute of Virology, Heinrich Heine University, Düsseldorf, 40225 Germany; ^4^ Vitalant Research Institute, San Francisco, CA, 94118-4417 USA; ^5^ Veterans Administration Medical Center, San Francisco, CA, USA; ^6^ University of Georgia, Animal Health Research Center, Athens, GA, 28130 USA; ^7^ Santo Biotech, LLC, Pendleton, IN, USA; ^8^ MS Bioworks, Ann Arbor, MI, USA; ^9^ Department of Global Health, University of Washington, Seattle, WA, 98195, USA; ^10^ Onipro LLC, Kensington, CA, USA; ^11^ University of California, San Francisco, CA, 94143, USA; ^12^ Viral Special Pathogens Branch, US Centers for Disease Control and Prevention, Atlanta, GA, USA

**Keywords:** drug discovery, host–viral interface, phenotypic screen, pan-respiratory antiviral therapeutics, allosteric modulator, viral capsid assembly

## Abstract

We present a novel small molecule antiviral chemotype that was identified by an unconventional cell-free protein synthesis and assembly-based phenotypic screen for modulation of viral capsid assembly. Activity of PAV-431, a representative compound from the series, has been validated against infectious viruses in multiple cell culture models for all six families of viruses causing most respiratory diseases in humans. In animals, this chemotype has been demonstrated efficacious for porcine epidemic diarrhoea virus (a coronavirus) and respiratory syncytial virus (a paramyxovirus). PAV-431 is shown to bind to the protein 14-3-3, a known allosteric modulator. However, it only appears to target the small subset of 14-3-3 which is present in a dynamic multi-protein complex whose components include proteins implicated in viral life cycles and in innate immunity. The composition of this target multi-protein complex appears to be modified upon viral infection and largely restored by PAV-431 treatment. An advanced analog, PAV-104, is shown to be selective for the virally modified target, thereby avoiding host toxicity. Our findings suggest a new paradigm for understanding, and drugging, the host–virus interface, which leads to a new clinical therapeutic strategy for treatment of respiratory viral disease.

## Graphical abstract

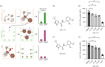

## 1. Background

The current SARS-CoV-2 pandemic has been characterized by waves of infection. Emerging mutants, with varying degrees of resistance to current vaccines and waning immune responses within the population, have contributed to the seemingly unending surges of disease [1,2]. Furthermore, the risk of a new pandemic, from avian influenza, respiratory syncytial virus (RSV) or another virulent pathogen known to exist in animal reservoirs, is ever present [3]. Given how rapidly SARS-CoV-2 spread across the globe once it had been transmitted to humans, concern about highly pathogenic respiratory viruses should not be considered as an abstract, hypothetical threat [4]. A technical solution is needed that can account for the degrees of uncertainty and variation inherent to pandemic preparedness and response efforts. Otherwise, antiviral countermeasures will continue to aim at an ever-moving target and always be one step behind. In this paper, we will propose a novel solution—one non-toxic small molecule compound with potent activity against all six families of viruses that cause most respiratory viral disease in humans.

Viruses in *Adenoviridae*, *Coronaviridae*, *Herpesviridae*, *Orthomyxoviridae*, *Paramyxoviridae* and *Picornaviridae* families cause over 95% of respiratory disease in humans [5]. The diversity between these viral families, which include both DNA and RNA viruses, and viruses that are both enveloped and not, is extremely broad [5]. The drugs that are available to treat some of these viruses target the varying proteins encoded by the different viral genomes [6–8]. Oseltamivir (Tamiflu) and zanamivir (Relenza) work on influenza by inhibiting neuraminidase, a viral enzyme that propagates infection by facilitating the spread of viral particles throughout the host [8]. Acyclovir, a treatment for herpes simplex virus, inhibits viral DNA polymerase [6]. Nirmatrelvir, the major component of Paxlovid, the new drug for SARS-CoV-2, is a protease inhibitor that blocks viral enzymes responsible for catalysing critical maturation steps within the virus's life cycle [7]. But since any one of these viral families represents a small minority of respiratory viral cases, a diagnosis must be made before potentially effective treatment is initiated. Yet, considerable evidence suggests that the earlier treatment is initiated, the greater is its therapeutic efficacy [9].

Host-targeted antiviral drugs have been proposed as a new strategy for antiviral drug development [10–14]. Viruses can only reproduce successfully if they are able to redirect host machinery to meet viral needs (e.g. by building its capsid, blocking immune response, etc.) rather than the needs of the host, which is to maintain homeostasis [15]. The viral generation time is several orders of magnitude shorter than the host's, making it likely that the host–virus interactome has been highly selected by viral evolution to provide the best way to reprogram host machinery [16,17]. While viruses employ a range of strategies for hijacking host machinery, ‘high value’ sites of host–viral interface are likely to be exploited by more than one family of viruses. Those sites would make ideal targets for pan-family antiviral drugs, but identifying them is a challenge.

We hypothesized that it would be possible to identify these high-value host–viral interface sites and develop drugs that target them, using cell-free protein synthesis and assembly (CFPSA) systems [13,18,19]. Cell free systems have been used to observe and understand critical molecular-level processes since 1897 when Eduard Buchner demonstrated that cell-free extracts could carry out the same fermentation reactions as living cells [20]. More recently, cell-free protein synthesis has been a critical tool used to decipher the genetic code, deconvolute protein trafficking, and functionally reconstitute the transient virus–host–protein interactions that culminate in viral capsid formation [21–25]. The last of these applications, which gave rise to the observation that viral capsid assembly in the cell-free system is dependent on both host machinery and metabolic energy, provided the rationale for developing our antiviral drug screen. Our hypothesis was that if viral capsid assembly is a host-catalysed process, then antiviral therapeutics could be developed by inhibiting the critical host enzymes co-opted by a virus to catalyse assembly of its capsid. To test this hypothesis, we set up a phenotypic screen for compounds that could block viral capsid formation in the CFPSA system, without inhibiting protein synthesis [13,19].

There are several advantages of a CFPSA-based drug screen. First, it uniquely serves to magnify early events in protein biogenesis that would otherwise be obscured by events in the rest of a protein's life cycle within the cell. Second, it recreates the reality of protein heterogeneity in cells, including with respect to post-translational modifications (PTMs) [26–28] and multi-protein complex (MPC) formation [29–31]. Finally, it exploits the recent appreciation that critical events in protein-protein interactions may occur co-translationally, that is, while a protein is nascent [32–36]. While in principle such a screen could detect direct binders of the translated viral protein(s), we suspected that the effect of binding a catalytic host target would be much greater, since blocking one enzyme molecule affects many orders of magnitude more substrate molecules and, in this case, the viral capsid monomer would effectively be the substrate for the enzymes that catalyse assembly. Furthermore, if the drug binds to an allosteric site rather than an active site, the mode of action would be more physiologically relevant [37–39].

There is a presumption that drugs that target host proteins pose an inherent risk of toxicity [14]. However, one implication of the burgeoning literature on the occurance of ‘moonlighting’ functions of proteins is that only a small subset of any given protein is likely to participate in any particular MPC [40–42]. Once a hit compound was identified by the CFPSA screen it should then be possible to drive its structureactivity relationship (SAR) to selectivity for the relevant subset of the target protein. We therefore anticipated the need to defer full assessment of toxicity until after SAR advancement of initial hits. Once an antiviral compound targeting the host were identified by CFPSA, it could subsequently be advanced separately for efficacy and for moderation of toxicity. The latter could be achieved either by virtue of the target being a small subset of the full complement of the targeted protein in the cell, or if the virus modified the host target for its needs, such that SAR might be selectively tuned to the form of the target needed by the virus.

The results, to be provided in this paper, focus on the advancement of one novel chemical series identified as a viral assembly modulator in the CFPSA screen, that appears to show pan-family antiviral efficacy in cells and animals. Experiments seeking to advance our antiviral chemical series and better understand its target and mechanism of action provide a new understanding of the host–viral interface and demonstrate a path, through SAR advancement, to next-generation antiviral therapeutics that selectively target the virally modified subset of a host protein, and thereby substantially avoid host toxicity.

## 2. Results

### 2.1. Identification and assessment of early assembly-modulating hit compounds PAV-773 and PAV-835

**Figure 1.**
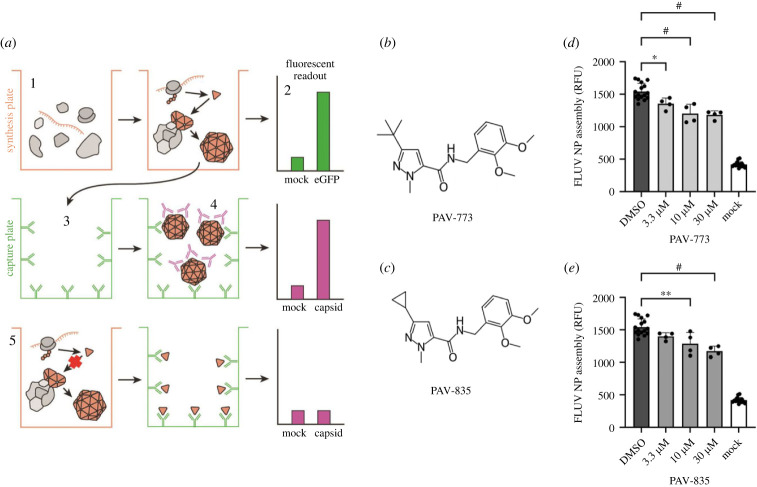
Identification of PAV-773 and PAV-835 as FLUV assembly inhibitors. (*a*) Schematic of the CFPSA phenotypic drug screen indicating steps and readouts. CFPSA reactions carried out in a 384 well plate format **(1)** result in synthesis of encoded FLUV proteins, with co-expression of eGFP to distinguish compounds that lower fluorescence readout due to a trivial effect on protein synthesis **(2)**. Assembled products are transferred to a capture plate **(3)** which is coated with antibodies to the FLUV nucleoprotein, capturing and immobilizing newly synthesized FLUV nucleoprotein. As a function of multimerization, unoccupied epitopes will exist on the antibody bound assembly Intermediates and completed viral structures captured on the plate. A biotinylated version of the primary antibody can be used to then decorate the captured products via those unocupied epitope enabling a fluorescence readout via avidin-horseradish peroxidase (HRP) binding to the biotin, washing, and addition of a fluorogenic HRP substrate such as Quanta Blue. **(4)**, generating a fluorescence readout of multimeric assembly. Drug action that directly or indirectly blocks multimer formation results in a diminution of fluorescent signal **(5)**. (*b*,*c*) Chemical structure of (*b*) PAV-773 and (*c*) PAV-835, early hits in the CFPSA screen. (*d*,*e*) Effects of (*d*) PAV-773 and (*e*) PAV-835 at 3.3, 10 and 30 µM doses on assembly of FLUV NP in the screen, compared to DMSO and a mock negative control. Average relative fluorescent units (RFU) detected from quadruplicate-repeat samples are graphed with standard deviation shown as error bars and statistical significance calculated on GraphPad Prism using an ordinary one-way ANOVA test is indicated by asterisks.

A cell-free protein synthesis and assembly (CFPSA) based phenotypic screen was established for influenza (FLUV) analogous to what has been done for rabies, HIV and other viruses [13,19,43,44]. Unlike conventional phenotypic screens, this screen was carried out in cellular extracts rather than in living cells. The phenotype being screened was the ability of newly synthesized viral capsid protein to form multimers culminating in structures indistinguishable from authentic capsids [22,23]. In the CFPSA system, faithful formation of multimeric capsid protein complexes is a quantifiable, functional endpoint (see diagram in figure 1*a*).

From a library of 150 000 drug-like small molecules, 30 400 compounds were screened and compounds that interfere with the biochemical pathway of host-catalysed FLUV capsid assembly were identified as hits. PAV-773 and PAV-835 were early compounds from such a chemical series identified in the screen as inhibitors of FLUV capsid assembly (see figure 1*b*,*c* for their respective chemical structures). Both compounds blocked assembly of FLUV nucleoprotein into a completed capsid in a dose-dependent manner, relative to control (see figure 1*d*,*e* for their respective activity against FLUV capsid assembly).

**Figure 2.**
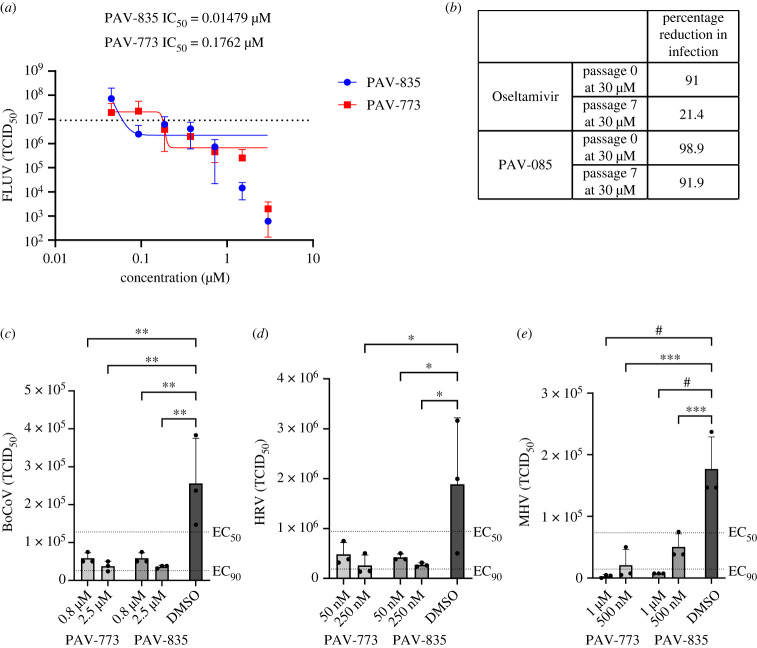
Validation of PAV-773 and PAV-835 antiviral activity in cell culture and evidence for a barrier to resistance development. (*a*) Activity of PAV-773 and PAV-835 against infectious FLUV (A/WSN/33; MOI = 0.01) in MDCK cells by TCID_50_ determination. Averages and standard error of triplicate samples are graphed, and EC_50_ values were calculated by GraphPad Prism. [Inhibitor] versus response – Variable slope (four parameters) was used to calculate the curves and EC_50_s. (*b*) Activity of PAV-835 or oseltamivir against FLUV (A/WSN/33; MOI = 0.01) in MDCK cells after 7 passages in the presence of compound. (*c*) Activity of PAV-773 and PAV-835 against infectious coronavirus (BRCV-OK-0514-2; MOI = 1) in HRT-I8G cells by TCID_50_ determination. (*d*) Activity of PAV-773 and PAV-835 against infectious rhinovirus (HRV-16; MOI = 5) in HI-HeLa cells by TCID_50_ determination. (*e*) Activity of PAV-773 and PAV-835 against infectious herpesvirus (MHV-68; MOI = 0.5) in BHK-21 cells by TCID_50_ determination. PAV-773 and PAV-835 both displayed EC_50_s of less than 1 µM for all four viruses studied. Statistical significance for (*c–e*) was calculated by ordinary one-way ANOVA tests on GraphPad Prism and is indicated with asterisks.

The FLUV antiviral activity of PAV-773 and PAV-835 was validated against infectious virus in MDCK cells by TCID_50_ determination (figure 2*a*). The effective concentration for half maximal activity (EC_50_) against infectious FLUV for both PAV-773 and PAV-835 were 177 and 42 nM, respectively.

The emergence of viral resistance is a common challenge for the development of effective antiviral therapeutics [45]. Oseltamivir (Tamiflu), an antiviral small molecule targeting FLUV neuraminidase, is known to select for viral resistance mutants [46]. To assess the propensity for FLUV to gain resistance to our chemotype, MDCK cells were infected with serial passages of FLUV in the presence of PAV-835. With each passage, the infected media was used to infect fresh MDCK cells. Higher concentrations of compound were added with each passage to drive resistance (93.5 nM to 3 µM). After 7 passages with compound, PAV-835 retained the same activity against FLUV as it did against a naive strain which had been passaged for 7 times without compound, demonstrating a barrier to the development of resistance (see figure 2*b*). In parallel, the same experiment was conducted using Oseltamivir (ranging from 935 nM to 30 µM), antiviral resistance developed and the compound lost activity by passage 7 (see figure 2*b*).

We counter-screened PAV-773 and PAV-835 for activity against other viral families by assessment of viral titre in cell culture by TCID_50_ (see figures 2*c–e*). Both compounds were found to have EC_50_s of less than 1 µM against bovine coronavirus (BoCoV), human rhinovirus (HRV), and murine herpesvirus (MHV). These data led us to refer to these compounds as *pan-respiratory viral assembly modulators* based on their initial identification as modulators of FLUV capsid assembly and subsequent demonstration of efficacy against multiple respiratory disease-causing viruses.

### 2.2. Validation of the antiviral activity of PAV-773 and PAV-835 in animals

At the time we were characterizing the activity of these early compounds, an outbreak of porcine epidemic diarrhoea virus (PEDV) led to the loss of more than 10% of the pig population in the United States [47]. Since PEDV is a member of the coronavirus family, we predicted that while the chemical series was early in the drug-development process, the compounds would likely show antiviral activity against PEDV. PAV-773 and PAV-835 were assessed in outbred pigs randomized within each litter into control and treatment groups and infected with PEDV. Both compounds significantly increased the likelihood of survival, relative to the control (see electronic supplementary material, figure S1).

### 2.3. Characterizing the antiviral activity of PAV-431, a more advanced analog from the pan-respiratory assembly modulator chemical series

**Figure 3.**
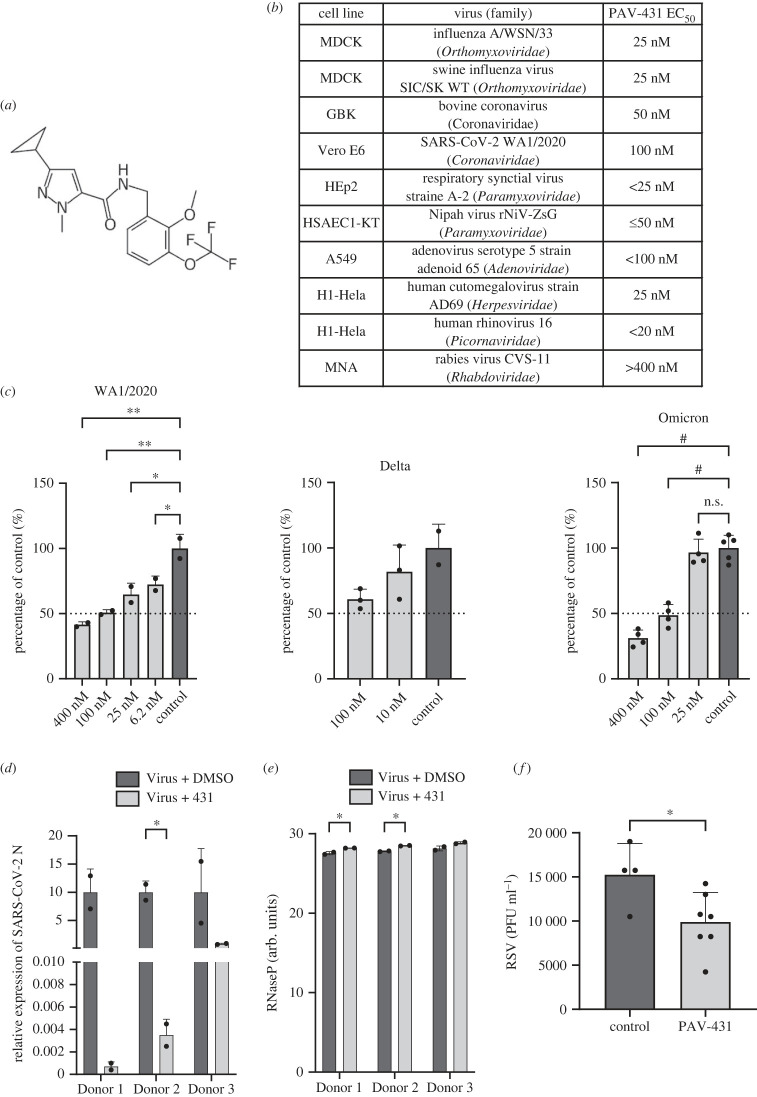
Antiviral activity of PAV-431 against the major viral families which cause respiratory disease in humans. (*a*) Chemical structure of PAV-431, an analog from the pan-respiratory assembly modulator chemical series. (*b*) Efficacy of PAV-431 against multiple viruses in cell culture by TCID_50_ where an EC_50_ of 100 nM or lower is observed for every family of virus causing human respiratory disease. (*c*) Dose-dependent antiviral activity of PAV-431 compared to a DMSO control against multiple SARS-CoV-2 strains: (Wa/2020, lineage A; MOI = 0.01) in Vero E6 cells, determined by plaque assay, delta variant (lineage B.1.617.2; MOI 0.01) and Omicron variant (lineage B.A.1; MOI 0.05) in Calu-3 cells determined by qPCR of the SARS-CoV-2 N gene and/or TCID_50_. Data shown are the averages of three biological replicates where error bars indicate standard error. Statistical significance was calculated on GraphPad Prism using an ordinary one-way ANOVA test for each dataset. (*d*,*e*) Efficacy and nontoxicity of PAV-431 in primary human airway epithelial cells at air-liquid interface. Bronchial epithelial cells from three lung transplant donors were culture to an air-liquid interface, infected with SARS-CoV-2 (gamma variant, lineage P.1; MOI = 0.1) and treated with either PAV-431 or vehicle. (*d*) Average of two replicates with error bars indicating standard error where viral replication was determined by qPCR measurement of the SARS-CoV-2N gene. (*e*) Lack of observed toxicity assessed by levels of RNase P. (*f*) Results of PAV-431 in an animal efficacy trial against RSV in cotton rats. Averages are shown where error bars indicate standard error. A significant drop in viral titre was observed with PAV-431 treatment, relative to vehicle (unpaired *t*-test *p* = 0.016). The statistical significance for (*d*–*f*) was calculated on GraphPad Prism using unpaired *t*-tests.

An SAR was pursued to advance the pan-respiratory assembly modulator chemical series emerging from the early hits and to understand how changes to the chemical structure altered activity against infectious FLUV (see electronic supplementary material, figure S2). PAV-431 was identified as a chemical analog with improved efficacy (figure 3*a* for its chemical structure and electronic supplementary material, figure S3*a* for its synthetic scheme).

PAV-431 was assessed by TCID_50_ for activity against multiple viral families. PAV-431 displayed an EC_50_ between 25 and 100 nM (depending on the virus) against members of *Orthomyxoviridae*, *Coronaviridae*, *Paramyxoviridae*, *Adenoviridae*, *Herpesviridae* and *Picornaviridae*—all six families of viruses which cause respiratory disease in humans (see figure 3*b*). Also within *Coronaviridae*, 100 nM of PAV-431 showed significant efficacy against the WA 1/2020 and Omicron strains of SARS CoV-2 as well as a clear trend with the delta variant (figure 3*c*). Finally, PAV-431 was assessed against Nipah virus, a BSL-4 member of the *Paramyxoviridae* family with pandemic potential should it ever jump species and become capable of human-to-human aerosol transmission, and shown comparably potent (see electronic supplementary material, figure S4).

In addition to demonstrating efficacy in transformed cells, PAV-431 showed activity against the gamma variant of SARS CoV-2 in primary human bronchial epithelial cells cultured to an air-liquid interface (ALI) (see figure 3*d*). To demonstrate antiviral activity of PAV-431 in these primary cells we measured the expression of the SARS-CoV-2N gene by qPCR. PAV-431 eliminated approximately 90% or more of viral load compared to vehicle treatment in three ALI studies derived from three different human lung donors without inflicting significant toxicity to the cells, as measured by levels of RNASe P (see figure 3*d*,*e*). Notably, PAV-431 did not show significant activity against rabies virus, indicating that even though the compound displays broad respiratory viral family efficacy, there is some selectivity for a target used by some, but not all, viral families (see figure 3*b*).

**Figure 4.**
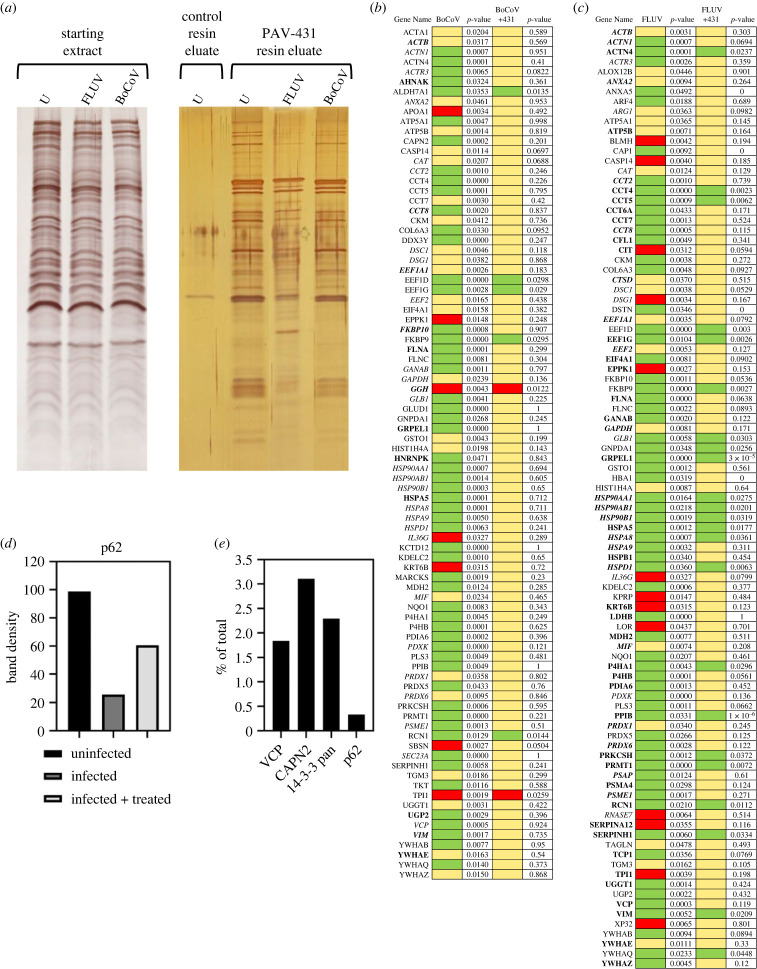
Protein composition of the PAV-431 eluate. eDRAC experiments were performed where uninfected, infected, or infected/PAV-431 treated cellular extract was incubated on a resin coupled to either PAV-431 or a 4% agarose matrix lacking the covalently bound drug. Infections were carried out with an MOI of 0.01. (*a*) Silver stain of an SDS-PAGE gel comparing protein composition of the starting cellular extract and the PAV-431 eluate for uninfected, FLUV infected and BoCOV infected MRC5 cells. (*b*) MS-MS analysis indicating protein composition and comparing log_2_-fold change and *p*-values in protein in triplicate repeated uninfected, FLUV infected, and FLUV/PAV-431 treated conditions. (*c*) MSMS analysis indicating protein composition and comparing log2 -fold change in protein in triplicate-repeated uninfected, BoCoV infected and BoCoV infected/PAV-431 treated conditions. Green indicates log2 fold change greater than 1. Yellow indicates log2 fold change between −1 and 1 (no change). Red indicates log2 fold change greater than −1. *p*-values indicate significance of the findings. Where the gene product has been listed in bold font, indicates the protein is implicated in the literature as part of the host-virus interactome. Where the gene product has been listed in italic font, indicates the protein is implicated in the literature as related to innate immune system function. (*d*) Quantitation of the protein band detected by western blot analysis of the uninfected, infected, and FLUV infected/PAV-431 treated eluates for the protein p62/SQSTM1. (*e*) Quantitation of the protein band detected by western blot analysis of eDRAC from pig lung extract where starting extract and eluate were compared side-by-side and the amount of protein in the eluate is graphed as a percentage of the total amount of that protein present in the cell extract. Approximately 2% of the cellular VCP, 3% of the cellular CAPN2, 2.5% of the cellular 14-3-3 and 0.5% of the cellular p62 was found in the PAV-431 eluate.

We assessed the degree to which the chemical properties of PAV-431 meet the standard criteria for advancement as a drug candidate. PAV-431 displayed promising properties including being negative for hERG channel inhibition, and without substantial CEREP panel enzyme inhibition although potential off-target effects at high concentrations could be relevant for 5-HT2B- and sodium channel receptors (see electronic supplementary material, figure S5*b*). When administered to rats, a dose of 5 mg kg^−1^ administered intraperitoneally (IP) was found to be safe, reaching a concentration of 108 nM in plasma and 167 nM in lungs (see electronic supplementary material, figure S5*a*). The following findings were obtained from a pharmacokinetics comparison between the earlier compound PAV-431 and a more advanced compound of the same lead series, PAV-104 [48]. From the electronic supplementary material, figure S5*a*, it is evident that PAV-104 had a better PK profile than PAV-431. AUC_last_ and AUC_inf_ of PAV-104 dosed IP-5 mg kg^−1^ are 2.5 times greater than those of PAV-431. PAV-104 exhibits a C_max_ that is 5 times greater via IV and 7 times greater via IP route. Regarding half-life, PAV 431 has a t_1/2_ that is six times more than PAV-104 via the IV route, whereas PAV 104 has a t_1/2_ that is 1.4 times greater than PAV-431 via the IP route. Although PAV-431 has a better volume of distribution than PAV-104, both compounds have a quick clearing tendency. PAV-104 exhibits good bioavailability, a crucial metric, with 95% availability, while PAV-431 has fair availability with 59% availability. PAV-104 has a superior profile than PAV-431 when used as an oral route too. PAV-104, dosed at PO-20 mg kg^−1^, has an excellent PK profile, as shown in figure 4*a*, but the levels obtained at dose PO-5 mg kg^−1^ with PAV-431 were too low to analyse the PK properties.

Additionally, uptake studies with PAV-431 and PAV-104 were conducted to evaluate their distribution in critical organs and tissues, namely the brain, lungs and plasma. PAV-431 was found to have good distribution in brain and lungs (target organ), the advanced molecule PAV-104 has better distribution through both routes tested in lungs and does not accumulate in brain at all. Both early and advanced molecules have good distribution in plasma.

Given the respectable PK properties, PAV-431 was tested in cotton rats infected with respiratory syncytial virus (RSV), a paramyxovirus, to assess animal efficacy for a more advanced compound in the series against a second family of respiratory disease-causing viruses. The drug was administered one day before infection and therefore *in this animal model* represents a prophylactic approach. A small but statistically significant drop in RSV titre was observed with PAV-431 treatment, relative to the vehicle-only control (see figure 3*f*).

Based on the improvements in PK profile noted, PAV-104 is likely to be superior to PAV-431 in future animal efficacy studies. However, CEREP panel and hERG channel inhibition and Ames mutagenicity testing have not yet been carried out on PAV-104.

### 2.4. Identifying the molecular target of the Pan-respiratory assembly modulators

Since this pan-respiratory viral assembly modulator chemical series had been validated as significantly active in cellular or animal models for six respiratory viral families, we sought to understand the molecular target being acted upon by the compound in order to achieve such broad results. To identify the target, PAV-431 was coupled to an Affi-gel resin from a position on the molecule unrelated to its biological activity, based on SAR exploration. Once bound to a resin, it could serve as a target-binding ligand for energy-supplemented drug resin affinity chromatography (eDRAC) (see electronic supplementary material, figure S3*b* for synthetic scheme of a PAV-431 resin). For eDRAC, extracts were prepared from MRC-5 cells that were uninfected, infected with either FLUV or BoCoV, and treated with 400 nM PAV-431 or an equivalent amount of vehicle (DMSO). When eDRAC free drug eluates from the PAV-431 resins and the control resin were collected and analysed by silver stain compared to the starting extract, several striking observations were made. While the protein profile in the starting extracts for uninfected, FLUV, and BoCoV infected cells appeared similar, the PAV-431 resin eluates were strikingly different, with a protein pattern not observed for free drug eluates from control resin (that is, a resin lacking the drug as an affinity ligand, see figure 4*a*).

Triplicate-repeat samples of eDRAC eluates generated from MRC-5 cell extract were sent for analysis by tandem mass spectrometry (MS-MS) to determine their protein composition. To analyse the data, LFQ intensity values for proteins identified in each condition were measured and compared against each other to generate log2 fold change values for each protein and each combination of conditions to provide a clear description of the differences observed under treatment conditions. Of 64 proteins identified by LFQ as increased in eluates upon FLUV infection, 41 are restored to uninfected levels after treatment with PAV-431 (see figure 4*b*). All 13 proteins lost from eluates upon FLUV infection are restored to uninfected levels after treatment with PAV-431 (see figure 4*b*). Of 56 proteins found increased in eluates upon BoCoV infection, 51 are restored to uninfected levels after treatment with PAV-431 (see figure 4*c*). Of 7 proteins lost from eluates with BoCoV infection, 5 are restored to the uninfected levels after treatment with PAV-431 (see figure 4*c*).

Proteins found to be significantly enriched or depleted by infection and/or treatment were searched in databases for known virus–host interactions and implication in the innate immune system interactome and many such proteins were identified (see figure 4*b*–*d*) [49–54]. P62/SQSTM1, a regulator of innate immunity, was identified in the PAV-431 eluate by western blot. As with changes in protein composition observed by MS-MS, the amount of p62 decreased with FLUV infection but was restored with PAV-431 treatment (see figure 4*d*).

**Figure 5.**
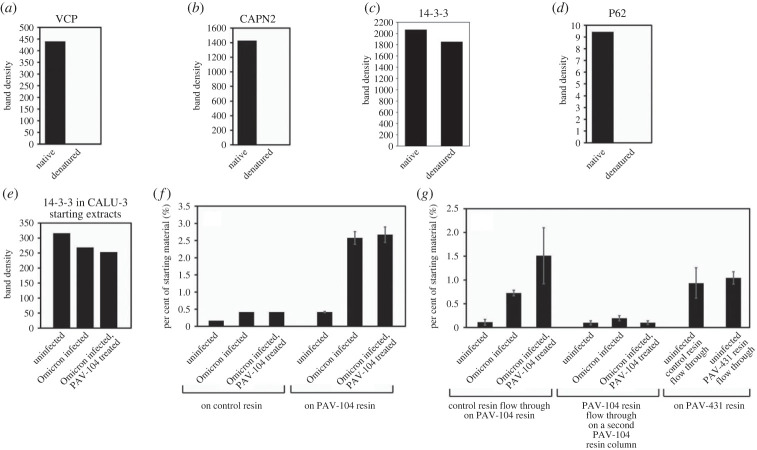
A cellular sub-fraction of 14-3-3 as the direct binding partner in an MPC drug target. eDRAC was conducted with pig lung homogenate extract eluted from the PAV-431 resin with the PAV-431 crosslinker analog. Eluates were exposed to UV light and precipitated with streptavidin in native and denaturing conditions then analysed by western blot. (*a–d*) Quantitation of the protein for VCP, CAPN2, 14-3-3 and p62. (*e–g*) eDRAC was conducted with uninfected, coronavirus (Omicron variant) infected, and coronavirus infected/PAV-104 treated cell extracts on the PAV-104 resin or a control resin where the unbound flow-through was run on a second resin. Infections were carried out with an MOI of 0.01. The diagrams show quantitation of the protein band by western blot for a 14-3-3 zeta (YWHAZ) antibody in the starting extract, bound to the first set of resins, and bound to the second set of resins. Graphs show the sum of the protein detected by western blot in both the eluate and the SDS strip, normalized as a percentage of the 14-3-3 zeta detected by western blot in the respective starting materials.

The eDRAC protocol was also conducted with extract prepared from uninfected pig lung homogenate, rather than MRC-5 cells, and samples were analysed by western blot. When analysed side-by-side with an aliquot of the total starting material, it was determined that for particular proteins found in the eluate including VCP, CAPN2, 14-3-3 and p62, only a single-digit percentage, or less, of the total amount of specific proteins present in the extract was found in the PAV-431 eluate (see figure 4*e*). The large majority of the component proteins did not bind to the resin, or bound nonspecifically such that they were removed with washing, with no significant further binding of drug resin flowthrough applied to a second copy of the drug resin (see also figure 5*g*).

To determine the relationship the proteins identified in the eluate had to one another, and to the compound, the eDRAC protocol was modified for photocrosslinking. An analog of PAV-431 was synthesized with diazirine and biotin moieties added to the same position at which the resin had previously been attached (see electronic supplementary material, figure S3*c* for synthetic scheme and chemical structure of photocrosslinker analogue). The photocrosslinker analogs were designed so that after an incubation with cell extract that would allow the compound to bind its target, exposure to ultraviolet light would form a covalent bond between the diazirine moiety of the compound and the nearest protein neighbor [55]. The sample could then be solubilized and precipitated with streptavidin beads (which bind biotin with extremely high affinity) to identify the covalently crosslinked drug-binding proteins. The streptavidin precipitation (SAP) could be done using a native sample, which would pick up the direct drug binding protein(s) and with it, co-associated proteins that were part of an MPC. Alternatively, the SAP could be done using a crosslinked sample that was then denatured by treatment with SDS to 1% and DTT to 1 mM with heating to 100°C for 3 min to denature all proteins, after which excess 1% Triton-X-100 buffer was added to take up free SDS into Triton micelles. Use of this material for SAP would, by virtue of the covalent bond to the biotin-containing diazirine-drug conjugate, identify only the direct drug-binding protein(s), with all other associated proteins lost upon denaturation and washing.

Uninfected pig lung 10 kxg/10 min supernatant was incubated on the PAV-431 resin under eDRAC conditions, washed 100 times, eluted with the PAV-431 crosslinker analog, then exposed to ultraviolet light. The samples were then divided into two equal parts where one was left native and the other denatured, then both were adjusted to non-denaturing conditions and incubated with streptavidin beads. Blots of the SAP samples for VCP, CAPN2 and p62 showed those proteins in the native but not denatured samples, indicating that they were non-covalently co-associated with the compound, and therefore were not its direct binding partner (see figure 5*a*,*b*,*d*). Blots of the SAP samples for 14-3-3 showed nearly equal amounts of protein in both the native and denatured conditions, indicating that PAV-431 directly binds to 14-3-3 (see figure 5*c*).

During the course of these studies, more advanced analogs of the lead series were synthesized. Studies presented elsewhere demonstrate that these advanced analogs, typified by PAV-104, are substantially more potent against SARS-CoV2 and less toxic than PAV-431 studied here [56,57]. Moreover, studies in mice indicated that PAV-104 was also substantially less toxic to animals despite its greater potency against virus in cell culture [56,57]. We hypothesized this to be due to PAV-104's selectivity for the virally-modified form of the host target, i.e. that present in infected extracts. To test this hypothesis, drug resin and photocrosslinker analogs matching those of PAV-431, were prepared for PAV-104. We also used these tools to extend to PAV-104 another observation made initially with PAV-431, namely that, while crosslinking data indicate that 14-3-3 is the direct target of PAV-431, the eDRAC data suggest this target comprises a very small subfraction of the total amount of 14-3-3 found in the cell (see figure 5*g*).

Extracts were prepared from uninfected, Omicron SARS-CoV-2 infected, and Omicron SARS-CoV-2 infected/PAV-104 treated Calu3 cells and used on the control and PAV-104 resins following the standard eDRAC procedure. The starting extracts were blotted for 14-3-3 and matched amounts of the protein were detected for all conditions, with slightly more in the uninfected (see figure 5*e*). When eDRAC was done using extract from uninfected cells, the 14-3-3 protein detected bound to the PAV-104 resin was comparable to that seen for the control resin (i.e. <0.5% of the total, see figure 5*f*). However, when eDRAC was done using extract from infected cells treated with either DMSO or PAV-104, 5x more 14-3-3 bound to the same resin (when normalized by the amount detected in the respective starting extracts, see figure 5*f*). To confirm the small percentage of 14-3-3 targeted by PAV-104 was specific as a drug target and not an artifact, the unbound flow through from the control resins and the PAV-104 resins were put onto a second PAV-104 column (see figure 5*g*). Compared to the flow through from the control resin no substantial amounts of 14-3-3 from the PAV-104 resin flow through bound to the second PAV-104 resin demonstrating that only a subfraction of total 14-3-3 within the cell extract binds to PAV-104 (figure 5*g*). However, when the flow through of the uninfected extract from the PAV-104 drug resin was run on a PAV-431 resin, a substantial amount of the 14-3-3 in the PAV-104 resin flowthrough bound to the PAV-431 resin. These results indicate that while both PAV-431 and PAV-104 specifically target very small subfractions of 14-3-3, the PAV-431 molecule targets a subset of 14-3-3 in uninfected cells which is *not* targeted by the advanced PAV-104 molecule (see figure 5*g*). It suggests that the advanced compound PAV-104, which is both an order of magnitude more potent and at least 3x less toxic (see electronic supplementary material, figure S5) appears to bind its 14-3-3 target primarily in extracts from infected rather than uninfected cells, even though there is ample 14-3-3 protein available in the uninfected extract. Thus, only a small subset of 14-3-3 is in the particular MPC drug target, and is not in equilibrium with the remaining >95% of total 14-3-3 in the cell.

## 3. Discussion

The antiviral chemotype studied here exhibits several notable features. These include (i) activity across a broad range of respiratory viral families, (ii) a demonstrated barrier to development of viral drug resistance and (iii) different forms of its target present in uninfected versus infected cells. In all cases, a small subset of the host protein 14-3-3, a protein known to work through allosteric interactions [58], appears to be the direct drug-binding protein and is present within a large MPC notable for its transience and energy-dependence. As part of a host–viral interface present in infected cells [56,57], the virally modified form of this target is preferentially bound by advanced analogs including PAV-104, while PAV-431 binds both forms of the target (i.e. that present in uninfected cells and that present in infected cells) roughly equally well. These compounds, with the unprecedented properties demonstrated, are advanced analogs of hits originating from the unconventional CFPSA screen described in figure 1.

Two general approaches can be taken for discovery of chemical compounds with therapeutic potential—target-based and phenotypic methods [59]. Target-based methods of drug discovery involve screens that measure a small molecule's interaction with a particular disease-implicated protein. However, the growing appreciation that proteins often act as part of an MPC [29] together with the burgeoning evidence [40,42] for protein ‘moonlighting’—multiple functions for a single protein—suggest this approach may miss many valuable drug targets. Phenotypic methods involve screens that monitor how small molecules affect particular biochemical or physiologic readouts within model systems without requiring any prior knowledge of the protein target. It has recently been observed that most drugs have been discovered by variations on phenotypic screening [60]. Most phenotypic screens involve whole-cell assays [61]. Such screens, while often successful, face significant drawbacks. The presence of confounding events in the complex milieu of a living cell can mask detection of potentially interesting targets. Moreover, feedback effects are typically complex and multifaceted [62–67]. This can create a signal-to-noise problem for detection of potential contributors to a particular phenotypic effect. Moreover, if multiple contributing factors are involved in creating a phenotype it may be hard to de-convolute the relationship of any given one to the behaviour of the compound.

By contrast, the CFPSA-based phenotypic screening approach taken here focuses attention on those events set into motion early in protein biogenesis (during and immediately after protein synthesis). This results in an improved signal-to-noise ratio by excluding much of the rest of the life cycle of most proteins, for both viruses and cells, as confounding variables. A growing literature supports the notion that protein assembly is co-translational, that is, occurs when at least one of the proteins involved is a nascent chain emerging from a ribosome [32,34]. Thus, a CFPSA-based screen may reveal aspects of the viral life cycle not easily discernable by other methods.

14-3-3, the protein identified as PAV-431's direct target (see figure 5) is known to regulate multiple signaling pathways, including cell cycle progression, apoptosis, autophagy, and glucose metabolism, through protein-protein interactions [58,68–71]. However, it has been difficult to convert these insights on 14-3-3 biology into therapeutic successes, perhaps because of this seeming ‘promiscuity’ of 14-3-3 [58,70–77]. Our data suggests a very different explanation for the difficulty in drugging 14-3-3 which is overcome by the methods shown here.

The 14-3-3 targeting antiviral chemotype identified through CFPSA is promising precisely because it does *not* target all of 14-3-3, but rather a tiny subset found within a particular transient, energy-dependent MPC. For this reason, most 14-3-3 in the cell is not perturbed by these drugs. Furthermore, the advancement from PAV-431 to PAV-104 has driven the SAR to substantial selectivity for the MPC present exclusively in infected cells. The data from eDRAC experiments provides compelling evidence that the 14-3-3 targeted by PAV-431 comprises only a single-digit percentage of the total amount of 14-3-3 present in the cellular extract (see figures 4 and 5). The data from photocrosslinking experiments provides evidence that this targeted subfraction of 14-3-3 is present in an MPC (see figures 4 and 5). PAV-431 was determined to directly bind 14-3-3 but it also was found to indirectly bind multiple other proteins including p62/SQSTM1, VCP, and CAPN2 that are present in the MPC (see figure 5*a–d*). Since 14-3-3 is known to regulate an array of cellular functions, data showing that PAV-431 targets a particular MPC provides a plausible explanation for why some but not all functions of 14-3-3 are regulated by the compound. The selectivity of PAV-431 to a small subset of 14-3-3 that is specific to a particular MPC or biochemical pathway makes the possibility of developing the chemical series as a 14-3-3 targeting therapeutic increasingly viable.

**Figure 6.**
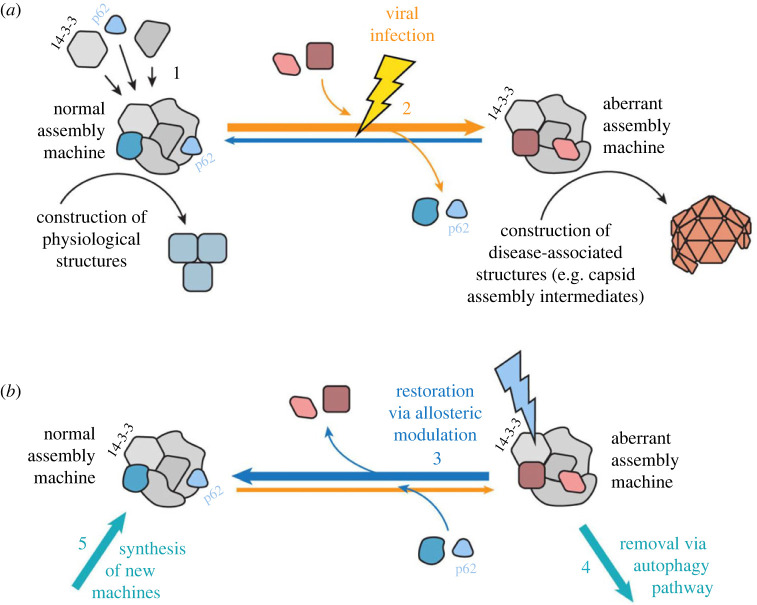
Cartoon diagram of proposed mechanism of action of assembly modulating compounds. (*a*) The proposed model where a ‘normal’ MPC with catalytic activity that plays a role in carrying out cellular events in the service of homeostasis is modified to an ‘aberrant’ MPC by a viral infection. The aberrant MPC carries out a reaction which does not serve homeostasis (e.g. is instead engaged in building a viral capsid) and perhaps fails to conduct a key event that it should (e.g. inform innate immune mechanisms that the cell is under attack). (*b*) The proposed mechanism in which treatment with an assembly modulating compound, such as PAV-431, normalizes the MPC, including restoration of p62, thereby restoring its homeostatic functions. The protein 14-3-3 is included in the diagram because it is the protein which the compound appears to directly bind. Its known role as an allosteric regulator may provide insight into how this normalization is achieved.

A key observation that builds on this point is that activity and target-selectivity can be pursued in tandem. PAV-104, an advanced analog of PAV-431 that displays significant improvements to anti-SARS virus activity and safety, targets an even smaller subfraction of 14-3-3 than does PAV-431 (see figure 6*f*,*g*). The flow through experiments show that PAV-104 targets specifically this tiny subfraction because when depleted material is passed onto a second PAV-104 resin, it will not bind any more 14-3-3, even though over 95% of the 14-3-3 in the starting extract is still present (figure 6*g*). However, PAV-431 can bind to 14-3-3 from the PAV-104 depleted material— indicating that PAV-431 targets *more* cellular 14-3-3 than PAV-104 does (see figure 6*g*). Furthermore, the subset of 14-3-3 that binds to PAV-104 only appears to exist in infected cell extract and not in uninfected cell extract. By contrast, PAV-431 binds to 14-3-3 in both infected and uninfected extracts (see figure 6*g*). The existence of a version of the target that only exists in infected cells raises the possibility that host-targeted drugs working in this dimension of post-translational heterogeneity may be driven to non-toxicity by advancement of molecules that are increasingly selective for forms of the target that only exist in the disease state. It should be noted that PAV-104 showed comparable rather than superior activity compared to PAV-431 against Nipah virus infection, suggesting slightly different virus-dependent compositions of the MPC including 14-3-3 (electronic supplementary material, figure S4), or some other basis for viral family selectivity, within the context of a pan-respiratory viral family active drug.

We originally termed hit compounds identified by our CFPSA screen as ‘assembly modulators’ because they blocked the assembly of viral proteins. However, based on the eDRAC and photocrosslinking results we would propose a more nuanced model for understanding the mechanism of action of assembly modulators. Our data suggests that viral infection modifies a multi-protein complex of the host with catalytic activity, to serve multiple alternate needs for the virus. This includes both promoting viral propagation through capsid assembly, and blocking innate immune defenses by ejecting p62/SQSTM1 from the MPC, likely thereby impacting p62/SQSTM1-mediated autophagy (see figures 4 and 5). This model is supported by the changes observed to the MPC targeted by PAV-431 under different conditions, which indicate that the target MPC's composition is dynamic (see figure 4). When cells are infected by viruses, certain proteins appear to be recruited and others appear to be ejected from the targeted MPC. When infected cells are treated with PAV-431, the reverse happens and the protein composition of the MPC appears to be largely restored to what was observed in uninfected cells (figure 6).

The significance of 14-3-3 as the direct binding partner of PAV-431 may be found in its known roles as an allosteric modulator of protein-protein interactions [58] and in its known participation in host anti-viral defenses (figure 6*b*) [78]. This may also, at least in part, account for the antiviral activity observed for PAV-431 against six diverse families of viruses causing human respiratory disease. The drug-binding site within 14-3-3 may represent a ‘high value’ site which multiple viral families, reflecting diverse structures and life cycles, have found valuable and devised means to exploit over deep evolutionary time. Whether these diverse viruses do so by the same or different mechanisms remains to be determined.

The relationship between particular proteins that comprise the targeted MPC and 14-3-3 as the direct drug-binding partner is unknown besides the evidence that they are transiently co-associated, and the observation that many of these proteins are implicated in the literature as being part of disease-relevant protein-protein interactomes [49–52]. While more data is needed, the potential significance of these early results involving the PAV-431 drug target is underscored by the loss upon viral infection, and return upon drug-treatment of infected cells, of p62/SQSTM1, an important regulator of autophagy [53,54,69]. An inability to trigger innate immune responses after viral infection would be to the virus's benefit and the host's detriment. Conversely, restoration of this function would bolster the host's ability to fend off infection. Thus the compounds presented here appear to have a dual mechanism of action: blockade of viral replication (capsid assembly) and restoration of autophagy, a branch of the innate immune system. This may also explain why the effect of PAV-431 in the cotton rat model of RSV infection was only modest, as the study protocol assessed its contribution to only one of these two mechanisms of action. A limitation of our studies is, that we cannot directly show at which point in viral replication (or capsid assembly) the function of 14-3-3 is impaired for the virus. A direct involvement of 14-3-3 in viral capsid assembly has not yet been shown for respiratory viruses. On the other hand, an important role for 14-3-3 as a chaperone, consistent with the findings here, has recently been proposed [79]. Chaperones have been proposed to play an important role in viral capsid assembly [80]. The transient nature of the MPC, orchestrated around 14-3-3 could be a reason why this specific sub-fraction 14-3-3 has not been previously identified as a direct factor involved in capsid assembly.

The identification of the pan-respiratory assembly modulating chemotype described here was achieved through unconventional methods. Its novel mechanism of action remains poorly understood. However the antiviral activity of compounds from the series have been validated against infectious viruses in both cell culture and animals (see figures 2 and 3 and electronic supplementary material, figure S1). The cell culture studies include primary bronchial epithelial cells cultured at an air–liquid interface and infected with SARS-CoV-2, a model considered as the gold standard for translatability into human therapeutics [81], which have confirmed the antiviral potency of these compounds (see figure 3 and [48]). Animal studies validated efficacy for survival in an actual pig coronavirus disease and viral load reduction in the cotton rat model of RSV infection (see electronic supplementary material, figure S1 and figure 4). The path to develop this chemical series to a clinical drug-candidate and conducting IND enabling studies, IND filing and human clinical trials on the lead compound is straightforward, especially since advanced chemical analog displaying substantial improvements to both antiviral activity and animal safety have already been identified. If approved as a drug, the assembly modulating compounds presented here may have transformative implications for the treatment of respiratory viral disease, applicable to everything from seasonal FLUV, common ‘winter viruses’ (HRS, HRV, etc), emerging variants of SARS-Cov-2 and any other particularly virulent strains of respiratory disease-causing viruses.

## 4. Material and methods

### 4.1. Correspondence and materials availability

Further information and requests for resources and reagents should be directed to and will be fulfilled by the corresponding author V.R.L.

Use of unique compounds PAV-431 and PAV-104 and their stable derivatives may be available upon request by the corresponding author if sought for experimental purposes under a valid completed Materials Transfer Agreement.

The number of replicates carried out for each experiment is described in the figure/table legends.

### 4.2. Chemical synthesis

#### 4.2.1. Synthesis of PAV-431

Synthetic schemes are illustrated in electronic supplementary material, figure S3. To a solution of 2-methoxy-3-trifluoromethoxy-benzaldehyde 1 (2.14 g, 9.71 mmol, 1.0 eq) in toluene (20 ml) was added 2,4-dimethoxybenzyl amine 2 (1.78 g, 10.68 mmol, 1.1 eq) and the reaction mixture was stirred at room temperature for 24 h. Toluene was removed to give a residue, which was taken in MeOH (20 ml) and then NaBH_4_ (735 mg, 19.42 mmol, 2.0 eq) was added slowly. The reaction mixture was stirred at room temperature for 6 h. The solvent was removed and the residue was extracted in ethyl acetate and stirred with saturated aq NaHCO_3_ for 1 h. The organic layer was collected, dried and the solvent was removed to give the crude amine 3, which was used in the next step without further purification. To a solution of the crude amine 3 (4.86 mmol, 1.0 eq) in DMF (20 ml) were added the acid 4 (888 mg, 5.35 mmol, 1.1 eq), DIEA (3.13 g, 24.3 mmol, 5 eq) and HBTU (2.22 g, 5.83 mmol, 1.2 eq) and the reaction mixture was stirred at room temperature for 12 h. The reaction mixture was then diluted with ethyl acetate (75 ml) and washed with 10% aq HCl (1 × 50 ml), sat NaHCO_3_ (1 × 50 ml) and water (4 × 50 ml). The organic layer was collected, dried (MgSO_4_) and evaporated to give a crude product, which was purified by column chromatography (EtOAc:Hexane 25:75%)) to give the amide 5, which was directly used in the next step. The amide 5 was treated with 95% TFA:H2O for 12 h. TFA was removed and azeotroped with toluene to give a residue, which was purified by column chromatography (EtOAc:Hexane 10:50%) to give PAV-431 (985 mg, >95% purity).

#### 4.2.2. Synthesis of PAV-431 resin

To a solution of amine 3 (5.85 g, 15.77 mmol, 1.0 eq) in DMF (30 ml) were added the acid 6 (2.38 g, 15.77 mmol, 1.0 eq), DIEA (10.2 g, 78.85 mmol, 5 eq) and HBTU (7.17 g, 18.92 mmol, 1.2 eq) and the reaction mixture was stirred at room temperature for 12 h. The reaction mixture was then diluted with ethyl acetate (75 ml) and washed with 10% aq HCl (1 × 50 ml), sat NaHCO_3_ (1 × 50 ml) and water (4 × 50 ml). The organic layer was collected, dried (MgSO_4_) and evaporated to give a crude product, which was purified by column chromatography (EtOAc/Hexane) to give compound 7. To a stirred solution compound 7 (0.8 g, 1.77 mmol, 1.0 eq) and cesium carbonate (1.15 g, 3.54 mmol, 2.0 eq) in DMF (10 ml) was added chloride 8 (0.55 g, 2.66 mmol, 1.5 eq) and the reaction mixture was stirred at room temperature for 24 h. The reaction mixture was diluted with ethyl acetate and washed with water (4×) and aq NaCl solution. The organic layer was collected, dried (MgSO_4_) and evaporated to give a crude product, which was purified by column chromatography (EtOAc/Hexane) to give compound 9. The amide 9 (1.0 g, 1.6 mmol) was taken in 95% TFA: H2O and the reaction mixture was for 12 h. TFA was removed and azeotroped with toluene to give a residue. The residue was taken in DCM and sat. NaHCO3 solution was added and stirred for 30 min. The aqueous layer was washed with DCM (2x) and the combined organic layer, dried (MgSO_4_) and evaporated to give a crude amine, which was used in the next step without purification. To a solution of the crude amine (1.6 mmol, 1.0 eq) and DIEA (412.8 mg, 3.2 mmol, 2.0 eq) in DCM (20 ml), was added boc anhydride (523.2 mg, 2.4 mmol, 1.5 eq) and the reaction mixture was stirred at room temperature for 8 h. The solvent was removed and the residue was purified by column chromatography (EtOAc/Hexane) to give compound 10. Compound 10 (100 mg, 0.19 mmol) was in 5 ml of DCM and then 4 M HCl in dioxane (3 ml, 12 mmol) was added and the reaction mixture was stirred for 12 h. Solvents were removed to give compound 11 as a HCl salt, which was used in the next step without further purification. To a solution of Affi-Gel 10 (Bio-Rad, 2 ml, 0.03 mmol, 1.0 eq) in a solid phase synthesis tube with frit was added a solution of compound 11 (27.7 mg, 0.06 mmol, 2.0 eq) and DIEA (1.0 ml) in isopropyl alcohol (4 ml) and the tube was put in a shaker for 12 h. Excess reagents were drained and the resin was washed with isopropyl alcohol (3×) and then saved in isopropyl alcohol.

#### 4.2.3. Synthesis of PAV-431 photocrosslinker

To 6-(tert-butoxycarbonylamino)-2-(9H-fluoren-9-ylmethoxycarbonylamino)hexanoic acid 12 [468 mg (1 mmol)] in a 40 ml screw top vial was added 4 N HCl in Dioxane (3 ml). The vial was sealed and gently agitated for 20 min at room temperature. The mix was then rotary evaporated to dryness and the residue was placed under high vacuum overnight. The dried residue was taken up into 4 ml of DMF (anhydrous) and then sequentially treated with 3-(3-Methyldiazirin-3-yl)propanoic acid [128 mg (1 mmol)](42), and DIEA [695ul (4 mmol)]. With rapid stirring, under Argon atmosphere, was added dropwise HATU [380 mg (1 mmol)] dissolved in 1 ml of DMF. After stirring for 30 min the mixture was quenched with 10 ml of sat. NH4Cl solution and then extracted 2× with 10 ml of EtOAc. The combined organic extracts were washed once with sat. NaCl, dried (Mg2SO4) and then rotary evaporated to dryness. The residue was purified by flash chromatography, using a gradient of Ethyl acetate and Hexane, affording 2-(9H-fluoren-9-ylmethoxycarbonylamino)-6-[3-(3-methyldiazirin-3-yl)propanoylamino]hexanoic acid 13 (293 mg) in 61% yield.

Amine 11 was taken up into 1 ml of DMF (anhydrous) and then sequentially treated with compound 13 [14.5 mg (0.03 mmol)], and DIEA [32ul (0.18 mmol)]. With rapid stirring, under Argon atmosphere, was added dropwise HATU [14.6 mg (0.038 mmol)] dissolved in 300ul of DMF. After stirring for 30 min the mixture was quenched with 5 ml of sat. NH4Cl solution and then extracted 2× with 5 ml of EtOAc.

The combined organic extracts were washed once with sat. NaCl, dried (Mg2SO4) and then rotary evaporated to dryness. The residue was purified by flash chromatography, using a gradient of ethyl acetate and hexane, affording 9H-fluoren-9-ylmethyl N-[1-[3-[3-cyclopropyl-5-[[2-methoxy-3-(trifluoromethoxy)phenyl]methylcarbamoyl]pyrazol-1-yl]propyl-methyl-carbamoyl]-5-[3-(3-methyldiazirin-3-yl)propanoylamino]pentyl]carbamate 14 (28 mg) in quantitative yield.

To compound 14 [28 mg (0.03 mmol)] in a 40 ml screw top vial was added 50/50 Diethylamine/DMF (0.5 ml). The vial was sealed and gently agitated for 60 min at room temperature. The mix was then rotary evaporated to dryness and the residue placed on high vacuum overnight. The residue was triturated 2× with 3 ml of Hexane to remove the Dibenzofulvene amine adduct. The residue was again briefly placed on high vacuum to remove traces of Hexane. The dried residue was taken up into 1 ml of DMF (anhydrous) and then treated with Biotin-PEG2-NHS [15 mg (0.03 mmol)] (purchased from ChemPep), and DIEA [16ul (0.09 mmol)] and then purged with Argon. After stirring overnight at room temperature, the mixture was rotary evaporated to dryness. The residue was purified by reverse phase prep chromatography, using a gradient of 0.1% TFA water and Acetonitrile, affording 5-cyclopropyl-N-[[2-methoxy-3-(trifluoromethoxy)phenyl]methyl]-2-[3-[methyl-[6-[3-(3-methyldiazirin-3-yl)propanoylamino]-2-[3-[2-[2-[5-(2-oxo-1,3,3a,4,6,6a-hexahydrothieno[3,4-d]imidazol-4-yl)pentanoylamino]ethoxy]ethoxy]propanoylamino]hexanoyl]amino]propyl]pyrazole-3-carboxamide (26 mg) in 80% yield. All compounds were confirmed by LCMS.

## 5. Method and analysis details

### 5.1. *In vitro* studies

#### 5.1.1. CFPSA screen

Coding regions of interest were engineered behind the SP6 bacteriophage promoter and the *Xenopus* globin 5′ UTR [82]. DNA was amplified by PCR and then transcribed *in vitro* to generate mRNA encoding each full-length protein. Translations were carried out in wheat germ extracts supplemented with energy and amino acids, as previously described (7). Moderate-throughput small molecule screening was carried out in 384-well plate format by translation of eGFP and FLUV NP and M mRNA in the presence of small molecules from the Prosetta compound collection (electronic supplementary material, figure S2). Reactions were run at 26°C for 1–2 h for synthesis, followed by assembly at 34°C for 2 h. eGFP fluorescent readout was measured at 488/515 nm (excitation/emission) to assess protein synthesis. Assembly products were captured on a second 384-well plate precoated with affinity-purified FLUV NP antibody. Plates were washed with PBS containing 1% Triton X-100, decorated with biotinylated affinity-purified FLUV NP antibody, washed, detected by NeutraAvidin HRP, washed again and then incubated with a fluorogenic HRP substrate Quanta Blue for 1 h. FLUV assembly fluorescent readout was measured at 330/425 nm (excitation/emission).

#### 5.1.2. FLUV assay in MDCK cells

MDCK.2 cells were seeded at 3 × 10^4^ cells per well in Eagle's minimal essential medium (MEM) supplemented with fetal bovine serum (FBS) in a 96-well plate and incubated overnight at 37°C. The next day, cells were washed with phosphate-buffered saline (PBS) and infected with FLUV A/WSN/33 at an MOI of 0.01–0.001 for 1 h, after which the virus-containing media was removed and fresh media containing dilutions of compound or DMSO as a vehicle control was added to the cells. After 24 h, media was removed, cells were washed with PBS, and fresh media was added for a 2 h incubation and then collected for TCID_50_ determination. Seven replicates of 10-fold serial dilutions of collected media were added to new cells and incubated at 37°C for 3 days. The number of infected wells for each dilution was determined by visual inspection, and TCID_50_/mL was calculated using the Reed and Muench method. Infection experiments were conducted in a BSL2 laboratory.

#### 5.1.3. BoCoV assay in HRT-18G cells

HRT-18G cells were seeded at 3 × 10^4^ cells per well in Dulbecco's modified Eagle medium (DMEM) in a 96-well plate and incubated overnight at 37°C. The next day, cells were infected with BoCoV BRCV-OK-0514-2 (ATCC VR-2460) at an MOI of 1 for 2 h, after which the virus-containing media was removed, cells were washed with PBS, and fresh media containing dilutions of compound or DMSO as a vehicle control was added to the cells. After 42–48 h, media was removed, cells were washed with PBS, and fresh media was added for a 4 h incubation and then collected for TCID_50_ determination. Infection experiments were conducted in a BSL2 laboratory.

#### 5.1.4. HRV assay in H1-HeLa cells

H1-HeLa cells were seeded at 7 × 10^4^ cells per well in MEM in a 96-well plate and incubated overnight at 37°C. The next day, cells were infected with HRV-16 at an MOI of 5 for 1.5 h, after which the virus containing media was removed, cells were washed with PBS, and fresh media containing dilutions of compound or DMSO as a vehicle control was added to the cells. After 72 h, media was collected for TCID_50_ determination. Infection experiments were conducted in a BSL2 laboratory.

#### 5.1.5. MHV assay in BHK-21 cells

BHK-21 cells were seeded at 2.5 × 10^5^ cells per well in MEM in a 96-well plate and incubated overnight at 37°C. The next day, cells were infected with MHV-68 at an MOI of 0.5 for 1.5–2 h, after which the virus-containing media was removed, cells were washed with PBS, and fresh media containing dilutions of compound or DMSO as a vehicle control was added to the cells. After 24 h, media was removed, cells were washed with PBS, and fresh media was added for a 4 h incubation and then collected for TCID_50_ determination. Infection experiments were conducted in a BSL2 laboratory.

#### 5.1.6. SARS-CoV-2 assay in vero cells

Vero clone E6 (CRL-1586) cells were plated at 3 × 10^5^ cells per well in DMEM in 12-well plates and incubated overnight at 37°C. The next day, cells were washed once with PBS and then infected with SARS-CoV-2 WA1/2020 (MN985325.1, BEI resources) at a MOI of 0.01 for 1 h after which the virus-containing media was removed. The compounds were mixed with overlay media consisting of 1.2% avicel/DMEM. The mix was then added to the cells and incubated for 72 h at 37°C at 5% CO_2_. The cells were then fixed and stained with crystal violet to determine plaque numbers [83] Infection experiments were conducted in a BSL3 laboratory. Data shown in figure 4*b* are the averages of two biological replicates; error bars indicate standard error; DMSO is included as the vehicle control.

#### 5.1.7. SARS-CoV-2 assay in Calu-3 cells

Calu-3 cells were seeded at a density of 3 × 10^4^ cells per well in DMEM in 96-well plates and incubated overnight at 37°C. The next day, cells were pre-incubated with compounds for 4 h before they were infected with SARS-CoV-2 delta SL102 (EPI_ISL_4471559) at a MOI of 0.01-0.05. After 24 h the viruses within 50 µl of the supernatants were lysed with 200 µl AVL-buffer (Qiagen) and 200 µl 100% ethanol was added for complete inactivation. RNA was extracted from 200 µl of the lysates using the EZ1 Virus Mini-Kit (Qiagen), and analysed by qPCR as described [39]. Infection experiments were conducted in a BSL3 laboratory. Data shown are the averages of three biological replicates; error bars indicate standard error; DMSO is included as the vehicle control.

#### 5.1.8. Recombinant ZsGreen-expressing Nipah virus infection

HSAEC1-KT cells were seeded at 10 000 cells per well the day prior to infection in 96-well black plates with clear bottoms (Costar 3603). The following day, cells were infected with recombinant Nipah virus expressing ZsGreen fluorescence protein (rNiV-ZsG) [84–87] at multiplicity of infection 0.01 with approximately 100 50% tissue culture infectious dose (TCID_50_). Levels of rNiV-ZsG replication were measured at 72 h post-infection based on mean ZsGreen fluorescence signal intensity (418ex/518em) using a Biotek HD1 Synergy instrument (Aglilent). Fluorescence signal intensity assayed in DMSO-treated, virus-infected cells were set as 100% ZsGreen fluorescence. Data points and error bars for all reporter assays indicate the mean value and standard deviation of four biological replicates, and are representative of at least 2 independent experiments in HSAEC1-KT cells. Concentrations of compound that inhibited 50% of the green fluorescence signal (EC_50_) were calculated from dose-response data fitted to the mean value of experiments performed for each concentration in the 10-point, 3-fold dilution series using a 4-parameter non-linear logistic regression curve with variable slope using GraphPad Prism 9 (GraphPad Software, La Jolla, CA, USA).

#### 5.1.9. Celltiter-Glo cell viability assay

Cell viability was assayed using Celltiter-Glo 2.0 assay reagent (Promega) according to manufacturer's recommendations, with luminescence measured at 72 h post-compound treatment using a Biotek HD1 Synergy instrument. Luminescence levels (indicative of cellular ATP levels as a surrogate marker of cell viability) assayed in DMSO-treated, uninfected cells were set as 100% cell viability. Dose response curves were fitted to the mean value of experiments performed for each concentration in the 10-point, 3-fold dilution series using a 4-parameter non-linear logistic regression curve with variable slope. All Celltiter-Glo cell viability assays were conducted in 96-well opaque white plates (Costar 3917). Concentrations of compound that inhibited 50% of the luminescence signal (CC50) were calculated from dose-response data fitted to the mean value of experiments performed for each concentration in the 10-point, 3-fold dilution series using a 4-parameter non-linear logistic regression curve with variable slope using GraphPad Prism 9 (GraphPad Software, La Jolla, CA, USA).

#### 5.1.10. Primary airway epithelial cell culture

Human bronchus was harvested from 3 explanted lungs. The tissue was submerged and agitated for 1 min in PBS with antibiotics and 5 mM dithiothreitol to wash and remove mucus. After 3 washes, the tissue was placed in DMEM with 0.1% protease and antibiotics overnight at 4°C. The next day the solution was agitated and remaining tissue was removed. Cells were centrifuged at 300 g/4°C for 5 min, then resuspended in 0.05% trypsin-EDTA and incubated for 5 min at 37°C. The trypsinization reaction was neutralized with 10% FBS in DMEM, then cells were filtered through a cell strainer and centrifuged at 300 g/4°C for 5 min. The cell pellet was resuspended in 10% FBS in DMEM and a 10 uL aliquot was stained with trypan-blue and counted on a hemocytometer. 7.5 × 10^4^ cells were plated onto each 6/0.4 mm FNC-coated Transwell air-liquid interface (ALI) insert. 10% FBS in DMEM and ALI media were added in equal volumes to each basal compartment and cultures were incubated at 37°C/5% CO2. The next day, media was removed and both compartments were washed with PBS and antibiotics. ALI media was then added to each basal compartment and changed every 3 days until cells were ready for use at day 28.

All studies involving SARS-CoV-2 infection of primary airway epithelial cells were conducted in the Vitalant Research Institute BSL3 High-Containment Facility. 6 h prior to infection, ALI medium containing dilutions of drugs (100 nM) or DMSO was added to the basal compartment. For infection, ALI medium containing drugs was removed, and SARS-CoV-2 diluted in ALI-culture medium containing drugs (100 nM, MOI = 0.1) was added on to the apical chamber of inserts (250 µl) and the basal compartment (500 µl). The cultures were incubated for 2 h at 37°C/5% CO2 to allow for virus entry, then washed, and 500 µl of fresh ALI medium containing drugs (100 nM) was added to the basal compartment. Drugs were maintained in the medium for the duration of the experiment. Cells were incubated at 37°C/5% CO2 and harvested for analysis at 36 h post-infection.

Total RNA was extracted from mock and SARS-CoV-2-infected primary airway epithelial cells with or without drug treatment lysed in Trizol (Thermo Fisher Scientific) using the chloroform-isopropanol-ethanol method. 500 ng of RNA was reversed transcribed into cDNA in 20 uL reaction volume using RevertAid First Strand cDNA Synthesis kit (Thermo Fisher) in accordance to the manufacturer's guidelines. RT-PCR was performed for each sample using TaqmanTM Universal Master Mix II, with UNG (Thermo Fisher) on the ViiA7 Real time PCR system. Primers and probes (2019-nCoV RUO kit) for detection of the SARS-CoV-2 Nucleocapsid (N) gene were obtained from IDT.

### 5.2. Alamar blue HS cell viability assay

Cell viability was assayed using Alamar Blue HS reagent (Thermofisher) according to manufacturer's recommendations, with fluorescence (560ex/590em) measured at 72 h post-compound treatment after 4 h of incubation with reagent using a Biotek HD1 Synergy instrument. Fluorescence levels (indicative of resazurin reduction as a surrogate marker of cell viability) assayed in DMSO-treated, uninfected cells were set as 100% cell viability. Dose-response curves were fitted to the mean value of experiments performed for each concentration in the 10-point, 3-fold dilution series using a 4-parameter non-linear logistic regression curve with variable slope. All Alamar Blue assays were conducted in 96-well black plates with clear bottoms. Concentrations of compound that inhibited 50% of the fluorescence signal (CC50) were calculated from dose-response data fitted to the mean value of experiments performed for each concentration in the 10-point, 3-fold dilution series using a 4-parameter non-linear logistic regression curve with variable slope using GraphPad Prism 9 (GraphPad Software, La Jolla, CA, USA).

### 5.3. Cell lysate preparation

Cells or tissues were extracted with PB buffer (10 mM Tris pH 7.6, 10 mM NaCl, 0.1 mM EDTA, and 0.35% Triton X-100), and centrifuged at 10 000 × g for 10 min. The supernatants were collected and flash frozen.

### 5.4. Energy-dependent drug resin affinity chromatography (eDRAC)

Drug resin was prepared by coupling compound PAV-431 to an Affi-gel resin at a concentration of 10 µM via the pyrazole nitrogen (electronic supplementary material, figure S3, synthetic chemistry described below), or position 4 of the phenyl group. Control resin was prepared by blocking the Affi-gel matrix without drug. Resins were equilibrated with column buffer (50 mM HEPES, pH 7.6, 100 mM KAc, 6 mM MgAc, 1 mM EDTA, 4 mM TGA) prior to any DRAC experiments. 30 µl of cell extract supplemented with energy (1 mM ATP, GTP, CTP and UTP with 4 mM creatine phosphate, and in some cases 5 µg ml^−1^ rabbit creatine kinase) was applied to resin columns. The columns were clamped and incubated at 22°C for 1 h for binding, and flow through was collected. The columns were then washed with 100 bed volumes of column buffer. For elution of bound complexes, 100 µl of column buffer containing free drug at a final concentration of 100 µM–1 mM (approaching its maximum solubility in water) and supplemented with energy was added, the column was clamped for 1 h, and serial eluates were collected. Eluates were analysed by SDS-PAGE and WB. or later use.

### 5.5. Western blotting

SDS-PAGE gels were transferred in Towbin buffer to a polyvinylidene fluoride membrane. Membranes were then blocked in 1% BSA, incubated for 1 h at room temperature in a 1:1000 dilution of 100 µg ml^−1^ affinity-purified primary antibody, washed three times in PBS with 0.1% Tween-20, incubated for 1 h in a 1 : 5000 dilution of secondary anti-rabbit or anti-mouse antibody coupled to alkaline phosphatase, washed further, and incubated in developer solution prepared from 100 µl of 7.5 mg ml^−1^ 5-bromo-4-chloro-3-indolyl phosphate dissolved in 60% dimethyl formamide (DMF) in water and 100 µl of 15 mg ml^−1^ nitro blue tetrazolium dissolved in 70% DMF in water, adjusted to 50 ml with 0.1 M Tris (pH 9.5)/0.1 mM magnesium chloride.

### 5.6. MS-MS analysis

Samples were processed by SDS-PAGE using a 10% Bis-Tris NuPAGE gel (Invitrogen) with the MES buffer system. The mobility region was excised and processed by in-gel digestion with trypsin using a ProGest robot (Digilab) with the protocol outlined below. Washed with 25 mM ammonium bicarbonate followed by acetonitrile. Reduced with 10 mM dithiothreitol at 60°C followed by alkylation with 50 mM iodoacetamide at room temperature. Digested with trypsin (Promega) at 37°C for 4 h. Quenched with formic acid, lyophilized, and reconstituted in 0.1% trifluoroacetic acid.

Half of each digested sample was analysed by nano LC-MS/MS with a Waters M-Class HPLC system interfaced to a ThermoFisher Fusion Lumos mass spectrometer. Peptides were loaded on a trapping column and eluted over a 75 µm analytical column at 350 nl min^−1^; both columns were packed with Luna C18 resin (Phenomenex). The mass spectrometer was operated in data-dependent mode, with the Orbitrap operating at 60 000 FWHM and 15 000 FWHM for MS and MS/MS respectively. APD was enabled and the instrument was run with a 3 s cycle for MS and MS/MS.

Data were searched using a local copy of Mascot (Matrix Science) with the following parameters. Enzyme: Trypsin/P; database: SwissProt Human plus the custom sequences (concatenated forward and reverse plus common contaminants); fixed modification: Carbamidomethyl (C); variable modifications: oxidation (M), acetyl (N-term), Pyro-Glu (N-term Q), deamidation (N/Q); mass values: monoisotopic; peptide mass tolerance: 10 ppm; fragment mass tolerance: 0.02 Da; max missed cleavages: 2. The data were analysed by label-free quantitation (LFQ) methods. LFQ intensity values of each condition were measured in triplicate and compared against each other to generate log2 fold change values for each protein and each combination of conditions. Proteins that were found significantly enriched by a log2 fold change of >1 and an adjusted *p*-value (accounting for multiple hypothesis testing) of <0.05 in the FLUV-infected eDRAC eluates compared to the uninfected eluates were searched for in a list of high-confidence FLUV virus-host protein interactions and the VirusMentha database of virus-protein interactions [49,50]. Likewise, significantly enriched and depleted proteins found in the BoCoV-infected eDRAC eluate were searched for in a list of high-confidence coronavirus interactors and an aggregated list of coronavirus protein interactors shown experimentally [51,52].

### 5.7. Photocrosslinking and streptavidin precipitation

eDRAC columns were eluted with 100 µM PAV-431 photocrosslinker at 22°C. Eluates were crosslinked by exposure to UV light for 3 min. Crosslinked products were subjected to treatments that maintained protein–protein associations (native) or which reduced and denatured all proteins (denatured). Native conditions were maintained by diluting an aliquot of the product 20x with 1% Triton-X-100 column buffer. Denaturation was achieved by adjusting an aliquot to 1% SDS and 10 mM DTT and heating to 100°C/10 min prior to 20× dilution with 1% Triton-X-100 column buffer. Streptavidin Sepharose beads were added to both native and denatured samples and mixed for 1 hr to capture all biotinylated proteins, with and without co-associated proteins in the native and denatured cases respectively, then washed 3× with 1% Triton-containing column buffer. Washed beads were resuspended in 20 µl of SDS loading buffer and analysed by SDS-PAGE and WB.

### 5.8. *In vivo* studies

#### 5.8.1. PEDV pig study

18 litters comprised of 91 individuals of newborn (2–4 days old) crossbred pigs weighing 3 kg were randomized to control (vehicle) or treatment groups. Animals were infected with 1 × 10^5^ PFU of PEDV administered orally. Vehicle or drug was administered intramuscular at 4 mg kg^−1^ immediately after challenge and again 24 h post-infection. Compound efficacy was determined by survivability. Endpoint of study was 6 days post-infection.

#### 5.8.2. RSV cotton rat study

Female cotton rats, approximately 5 weeks of age, were obtained from Envigo (formerly Harlan), ear-tagged for identification purposes, and allowed to acclimate for >1 week prior to study start. Animals were housed individually. Vehicle or drug (2 mg kg^−1^) was administered by an intraperitoneal route twice daily on study days −1 through day 4. On day 0, animals were infected with 1 × 10^5^ PFU of RSV A-2 virus originally obtained from ATCC (VR-1540), administered in a 50 µl volume by an intranasal route approximately 2 h after the morning treatment dose. Back titration of the viral stock and diluted inoculum was performed to confirm the titre of the RSV stock used for infection. All inoculations were performed while the animals were under the influence of inhalant anaesthesia. All animals were euthanized on day 5 and the lungs were processed for determination of RSV titres by plaque assay.

## Data Availability

Further information and requests for resources and reagents should be directed to V.R.L., corresponding author. Supplementary material is available online [88].
